# Kairos in diagnostics

**DOI:** 10.1007/s11017-023-09657-9

**Published:** 2024-02-07

**Authors:** Bjørn Hofmann, Urban Wiesing

**Affiliations:** 1 Centre of Medical Ethics, Faculty of Medicine, University of Oslo, PO Box 1130, Oslo, N-0318 Norway; 2 Institute for the Health Sciences, Norwegian University of Science and Technology (NTNU), Gjøvik, Norway; 3 Institute for Ethics and History of Medicine, University of Tübingen, Tübingen, Germany

**Keywords:** Diagnosis, Kairos, Chronos, Time

## Abstract

Kairos has been a key concept in medicine for millennia and is frequently understood as “the right time” in relation to treatment. In this study we scrutinize kairos in the context of diagnostics. This has become highly topical as technological developments have caused diagnostics to be performed ever earlier in the disease development. Detecting risk factors, precursors, and predictors of disease (in biomarkers, pre-disease, and pre-pre-disease) has resulted in too early diagnoses, i.e., overdiagnoses. Nonetheless, despite vast advances in science and technology, diagnoses also come too late. Accordingly, timing diagnostics right is crucial. In this article we start with giving a brief overview of the etymology and general use of the concepts of kairos and diagnosis. Then we delimit kairos in diagnostics by analysing “too early” and “too late” diagnosis and by scrutinizing various phases of diagnostics. This leads us to define kairos of diagnostics as the time when there is potential for sufficient information for making a diagnosis that is most helpful for the person. It allows us to conclude that kairos is as important in diagnostics as in therapeutics.

## Introduction

Kairos (καιρὸς) has been a key concept in medicine for millennia and is frequently understood as ‘the right time’ [1, p. 98]. It is prominently found in the first Hippocratic Aphorism: “ὁ δὲ καιρὸς ὀξύς”, “the right moment is fleeting”. The concept of kairos has mostly been referred to in medical treatment [2] or for disclosing “bad” news [3].

Nevertheless, the concept also is highly relevant in the context of diagnostics (not the least because technological developments have caused diagnostics to be performed ever earlier in disease development [4]). Detecting risk factors, precursors, and predictors of disease has resulted in “too early” diagnoses - e.g., when the diagnosis has no potential to lead to helpful consequences or when what is diagnosed will never develop into any health-related problems for the person in question [5]. In a time when a wide range of biomarkers and genetic variants of uncertain significance are identified, pre-disease is classified [6], and even pre-pre-disease (pre-pre-diabetes) is identified, [7] the question of when it is too early for diagnostics becomes pertinent. The same goes for (presymptomatic) conditions where no treatment can be offered (yet), such as Huntington’s disease. When is the right time to test and diagnose - if to test at all?

Additionally, despite vast advances in science and technology, diagnoses can also come too late. That means when the diagnosis is set, it is too late to offer the most helpful treatment. In the worst case, only palliative therapy options remain. Accordingly, there must be a “right time” for diagnostics, i.e., when it is neither “too early” nor “too late”.

Hence, while kairos has gained most attention in the context of therapy [1], we will concentrate on diagnostics. Accordingly, *the objective of this study is to scrutinize the kairos of diagnostics, i.e., the right time of diagnosing*. To address this issue, we start with a brief overview of the etymology and general use of the concepts of kairos and diagnosis. Then we analyse situations of “too early” and “too late” diagnostics in various phases of the diagnostic process in order to specify the kairos of diagnostics. Based on this we conclude that kairos is as important in diagnostics as in therapeutics.

## What does kairos mean?

The common definition of kairos is ‘the right time’ and ‘proportionate’ or ‘right measure’ [1, p. 98]. What is often meant by this is a very specific point in time, the right moment. There is also a reference to action in the concept of kairos; phrased as a ‘critical, or opportune moment’, and ‘a proper or opportune time for action’ [8]. To do something at “this time” means to do it at the “right time”.

Kairos is frequently contrasted with chronos (χρόνος) - meaning (chronological) time in its even course. The relationship between kairos and chronos in medicine was already addressed in antiquity. The famous quote in the Hippocratic writings about kairos is: ‘every kairos is a chronos, but not every chronos is a kairos’ [9, p. 98]. Kairos is therefore a selected time within chronos.

## What does diagnosis mean?

In order to study kairos in diagnostics, one also needs to have a clear concept of diagnosis. Etymologically, diagnosis stems from Greek diagignōskein - to distinguish or discern. The prefix dia means ‘apart’, ‘through’, and/or ‘thorough’, and gignōskein means ‘recognize’, ‘acknowledge’, and/or ‘know’ [10]. Hence, one etymological meaning of diagnosis is “to know thoroughly” and another is “discerning by knowledge”. In a practical medical context this frequently refers to gaining information and identifying (discerning) conditions by questioning and examining by the application of knowledge. Just as “conditions” have come to mean a wide range of things (biological, molecular, physiological, neurological, mental, etc.), the meaning of “information” has also been extended by a vast amount of paraclinical tests.

Moreover, there tends to be two different meanings of diagnosis that are not always distinguished in everyday language use. The term diagnosis can refer to a *disease entity* or a *diagnostic process*. That is: diagnosis can be a body of knowledge with different characteristics or a process of knowledge acquisition.

### Body of knowledge

If diagnosis is a body of knowledge, it is important to clarify whether it consists of the knowledge for assigning a disease entity to a patient, or of the sum of the knowledge relevant for medical actions. In modern (pragmatic) medicine, it is frequently the second option, the relevant knowledge for helpful action. This knowledge can also contain a disease entity but does not have to consist *only* of a disease entity. This is because medical actions do not only result from the assignment of a disease entity to a person, but also from further information that does not directly belong to the disease entity (e.g., from separate laboratory parameters or from the individual’s condition). In this understanding of diagnosis, “knowledge relevant for medical actions” refers to knowledge that has the potential to lead to helpful actions.

In this setting kairos can only refer to the second meaning of diagnosis, to a process, to *diagnosing*. While one can ask for the right time for setting a disease entity (or to convey a diagnosis), one cannot ask for the right time for a disease entity to manifest.

### Process

Diagnosing then means the process by which knowledge about the disease entity is sought. It is the knowledge-acquisition process which may end with a disease entity being attributed to a person. However, if diagnosis is the gathering of knowledge relevant to action, not just the attribution of a disease entity, then the question of this study may be specified in the following way: When is the right time to start diagnosing to acquire knowledge for further helpful decisions, and when does one have enough information to set a (temporary) diagnosis, i.e., when to finish the process (and make decisions/start acting)? Both starting and finalizing the diagnostic process can be too early or too late.

### Legitimation of diagnosis

Diagnosing is a key element of medical practice founded on medicine’s moral goal, i.e., to help individuals. Accordingly, the “right time” of diagnosis is when diagnostics contributes to the fulfilment of this ultimate goal.

Therefore, diagnosing is never an end in itself, but offers the chance to gather information that enables helpful action. While what counts as helpful may be broad, including offering explanation only, diagnoses must be beneficial. If it cannot be beneficial, it must not be used. The only exception to this rule is research, which in turn claims to increase the benefit to (other) patients (in the long term).

In summary, in the context of kairos, diagnosis means a process, the acquisition of knowledge for decisions to enable helpful actions. The kairos would then be the moment within this diagnosing process that is the most helpful to the individual. It is important to note that it is the potential for, not the certainty of, helpful information that justifies the reason to do diagnostics. Even if the diagnostics were performed completely correctly and in a timely manner, no helpful information may emerge; whereas it could have been possible for such information to emerge according to the knowledge that was available in the moment of starting diagnostics. Thus, a judgment on a diagnostic decision must take into account the knowledge that was available at the time the decision was made, not the knowledge after the diagnostic process was carried out.

## Kairos in diagnostics: between too early and too late

As pointed out, diagnostics can start too early [4] or too late [11–14] and can be finished too early or too late.

### Starting too early

*Too early diagnosis* can occur if diagnosing is commenced when no potential for helpful information exists. Too early diagnosis can be understood in two ways: First, the diagnostic procedures may be performed too early if there is no chance that diagnostically relevant information can be found at this time in the course of the disease (i.e., the diagnostic process was started too early). Second, relevant information may be found and used for a diagnosis and proposed interventions, but the intervention may have no benefits (as it was applied too early). Or, what was diagnosed would not have developed into something that would have been experienced negatively by the person (that is, the consequences of the diagnosis were inferred too early). This is normally called overdiagnosis [15]. While there is no compelling link between too early diagnosis and too early consequences, too early information tends to lead to premature action.

### Finishing too early

Finishing the diagnostic process too early means that further potentially helpful information could be gained at a later time. If diagnostics were finished too early helpful consequences could not be drawn as consequences from the diagnosis were inferred too early.

### Starting too late

*Too late diagnosis* would occur when diagnostics are started too late, delayed, missed, or are inadequate (i.e., when “the most helpful information” comes too late to be actionable). The consequences of too late diagnosis is often poorer prognosis (i.e., poorer outcomes in morbidity, mortality, and/or quality of life).

### Finishing too late

Finishing the diagnostics too late means that diagnostics were carried out that could not gather any additional relevant information for helpful consequences. As a result, helpful measures may be delayed as the diagnosis was set too late [13].

Accordingly, the general rule for the kairos of diagnostics can be understood as the time when there is the most potential for sufficient information for making a diagnosis that is most helpful for the health of a person.

## Kairos in different phases of diagnostics

In order to further analyse this “right time” for diagnosing, it is fruitful to scrutinise the various phases of diagnostics. There are “right timings” for testing, analysis/interpretation, conveying diagnosis, as well as receiving and accepting a diagnosis.

The right time for testing is when what is tested for and detected has a potential to matter to the person’s health [16]. The tests must be made at a time when they are most explanatory and/or actionable. Even more, the actions must matter for the person, and in particular for their health.

Correspondingly, the right time for analysis is when the interpretation of the results are most relevant to the person’s health. On the one hand, the analysis and interpretation of a genetic test may be outdated and may result in wrong decisions with harmful consequences, as has happened in Norway where 21 women unnecessarily had their breasts and ovaries removed based on outdated interpretations of genetic test results [17]. On the other hand, the interpretation or analysis may come too late to matter to the person’s health or worsen the condition and prognosis, potentially resulting in additional frustration.

The right time to convey the diagnosis is when the person can comprehend the diagnosis and its implications and deliberate based on the information about the diagnosis [18, 19]. Table 1 provides an overview of “too early”, “too late”, and the “right time” (kairos) of diagnostics for the various aspects and phases of diagnostics.

**Table 1:** Overview of too early, too late, and “timely diagnostics” (kairos) for various phases of diagnostics

	Too early	Kairos	Too late
Timing of information provision	No potential for sufficient information to make a warranted diagnosis	Potential at this time for sufficient information to make a warranted diagnosis	Information provided too late, ignored, or provided erroneously, resulting in lack of diagnosis
Testing	Overdiagnosis: finding things (conditions, indicators) that do not matter to the person’s health	What is tested for and detected matters to the person’s health	Underdiagnosis: finding things too late that matter to the person’s health
Analysis and interpretation of results	Analysis/interpretations based on incorrect or preliminary data (e.g., incorrect interpretation of genetic test results leading to inappropriate mastectomies and oophorectomies)	The interpretation of the results is made at a time when their results are valid and relevant for making qualified and beneficial decisions	Too late interpretation or analysis to matter to the person’s health (e.g., too late to prevent metastatic development of cancer)
Conveying diagnosis and Diagnosis reception	The person is not yet emotionally and cognitively able to comprehend and deliberate based on the diagnosis	The patient is at that time able to emotionally and cognitively comprehend and deliberate based on the diagnosis	The person is deprived of the opportunity to emotionally and cognitively comprehend and deliberate based on an early diagnosis but has to face with a worse situation and with poorer prognosis
Decisions based on diagnosis	There is too little information to make qualified and beneficial decisions based on the diagnosis	There is sufficient information to make qualified and beneficial decisions based on the diagnosis	It is too late to make optimal beneficial decisions based on the diagnosis
Prognosis based on diagnosis	Too early to make prognosis (i.e., inappropriate prognosis from inappropriate diagnosis)	Enough information at that time to make a qualified prognosis	The prognosis based on the overdue diagnostics is worse than the prognosis based on diagnostics of the right time

Importantly, there is a fundamental difference regarding possible consequences between “too early” and “too late diagnosis”: too early diagnosis does not necessarily lead to too early intervention. It is possible to let more time pass between the diagnosis and the intervention and then intervene at the optimal time. With a diagnosis that is too late, no temporal adjustments are possible. If diagnostic information is found too late for optimal therapy, then the time has passed, and this cannot be changed by further decisions. While sub-optimal consequences may be avoidable if the diagnosis is made too early, this may not be possible if the diagnosis is made too late.

### Specification of “the right time”

The kairos of diagnosis has been defined as the (period of) time when there is potential for sufficient information for making a diagnosis that is helpful for the person, existing between “too early” and “too late” diagnoses. However, the question remains: is kairos singular or plural? In view of the dynamism of the diagnostic process, are there several kairoi (καιροί)? The answer is “yes” as medical management is not just a one-time event, but a process in which questions about further diagnostics may arise more frequently, and thus also the question of “the right time”.

So, is kairos a moment or a period? If the question of the kairos applies to both the beginning and the completion of the diagnostic process, then there is indeed a period of time in between. Therefore, seeking kairos in diagnostics should not only ask about the right moment, but also about the right period of time. In other words, the question of kairos is not only when the right moment is, but how long it lasts. Consider that there can be substantial differences between emergency diagnostics, which usually must be made very quickly, diagnostics for chronic conditions, and testing/diagnostics for prevention decisions.

Another question persists - can kairos be graded or quantified? Is there kairos only in an ‘on/off’ sense, where kairos is either given or not given (had or not had), or are there more or less favourable moments in diagnosing? It is the latter; one moment in the diagnosing process can be comparatively more helpful than another. Indeed, these differences can be quantified with modern methods, e.g., digital clinical support systems or Artificial Intelligence [20]. However, should medicine, especially with the help of modern data processing, differentiate the right time in diagnostics in quantitative terms? Once again, the answer comes from the moral goal of medicine. If it could help the person, quantification would be warranted.

### The rule and the individual case

If the rule is that diagnostics are carried out at the right time when they contribute optimally to the patient’s benefit, then it is by no means trivial to determine this target point for an individual patient. This is because numerous individual factors flow into this judgment which must be taken into account. This is the well-known phenomenon that applying a rule to an individual case always requires professional judgment. One must always ask what the optimal time is in this specific case. Therefore, medical experience and careful judgment is needed.

## Discussion

Thus far, we have examined definitions of kairos and diagnosis to investigate what we call kairos in diagnostics, which has been defined as the right time for diagnosing, i.e., when it has a potential to gain sufficient information for contributing to the overall goal of medicine in terms of helping individuals. By studying “too early” and “too late” diagnostics, we have delimited the right time for various aspects of diagnosing, such as testing, analysing, interpreting, and conveying the diagnosis as well as making decisions and prognoses based on the diagnosis. Moreover, we have analysed kairos in terms of when to start the diagnostic process, the time of setting diagnosis, and the time of acting (e.g., with an intervention) based on the diagnosis.

By respecting timing in diagnostics, medicine can avoid underdiagnosis and overdiagnosis (and the negative outcomes from both of these), as well as frustration, anxiety, and uncertainty (in persons/patients), and reduced autonomy and poor deliberation. While biomarker development, precision and predictive medicine, artificial intelligence, machine learning, deep learning, and Big Data may reduce the probability of “too late diagnosis”, they may also increase the risk of “too early” diagnosis. Therefore, the kairos of diagnostics will remain ever relevant in and for the future.

Moreover, the issue of kairos in diagnostics touches upon basic ethical principles, such as beneficence, non-maleficence, justice, and autonomy. The principle of autonomy is especially relevant in terms of the right to know and not to know. If the diagnosis is made too early, the person has the right either not to know or to know about the uncertainty or preliminarity of the diagnosis. Correspondingly, if the diagnosis is made too late, resulting in prognostic shortfall, the patient has the right to know. However, the question of the right time to convey a diagnosis (“timely diagnosis”) is beyond the scope of this theoretical study and merits a separate examination.

While the issue of autonomy in kairos can be adapted to an individual’s preferences, beneficence in kairos can mostly be assessed on a population level [21] as false alarms, overdiagnosis, and other types of “too early” diagnosis. Unfortunately, “too early” diagnosis cannot be studied on an individual level (yet), as we do not know which indicators (precursors, risk factors, or predictors) will develop to anything harmful to the person [4].

Certainly, we have not been able to address all aspects of timing in diagnostics. For example, we have not defined *when* diagnostics has the “potential to provide sufficient information” as this is an issue of professional judgments and standards. However, we have addressed the main challenges in understanding the kairos of diagnostics, which is an important start.

Moreover, we have defined kairos as a process that provides sufficient information for making a diagnosis that is helpful for the person in terms of health, but we have not defined what is “helpful for the person” or what “health” is. We have chosen not to specify this, as it is a huge topic [22–24] that deserves its own study.

Another related aspect, which has not been discussed in this study, but which has been addressed elsewhere, is when to stop applying / remove a diagnosis, i.e., the right time to “de-diagnose”. The right time for removing a diagnosis is when there is no net benefit for the patient having the diagnosis [25].

## Conclusion

By investigating the concept of diagnosis and kairos, the kairos of diagnostics can be defined as the right time for diagnosing, i.e., when there is sufficient information for making a diagnosis that is helpful for the person in terms of health. It is therefore to be understood according to the overall goal of medicine and the context of medical practice. Since a diagnosis is not a temporal moment but can be a period in a process, the kairos of diagnosis can also be a period in a process. Accordingly, there is not only one kairos, but there can be multiple kairoi. Moreover, depending on how much it benefits the person, kairos can be graded or quantified.

The kairos of diagnostics exists between “too early: diagnosis (when, due to the timing, information is lacking, insufficient, or it does not benefit the person) and “too late” diagnosis (when, due to the timing, important information comes too late to consider relevant beneficial therapeutic interventions and leads to a poorer outcome in terms of morbidity, mortality, quality of life, and functional status). Moreover, the kairos of diagnostics can be specified for the various phases of diagnosing, such as testing, analyzing, interpreting, and conveying the diagnosis. In conclusion then, timing is as important in diagnostics as in therapeutics.
